# 258. Comparison of Different COVID Waves during COVID-19 Pandemic: A Retrospective Study from a Dedicated COVID-19 Facility of Karachi Pakistan

**DOI:** 10.1093/ofid/ofac492.336

**Published:** 2022-12-15

**Authors:** Muneeba A Sayeed

**Affiliations:** Sindh Infectious Diseases Hospital & Research Center/Dow University of Health Sciences, Karachi, Sindh, Pakistan

## Abstract

**Background:**

In Pakistan, the first case of COVID-19 was reported in February2020. To date Pakistan has experienced 5 waves of COVID-19 and each COVID-19 wave is driven by a unique SARS CoV2 strain. In this study we have compared the clinical spectrum and outcome of different COVID-19 waves in a dedicated COVID-19 facility of Karachi.

**Methods:**

We conducted a retrospective study at the Sindh Infectious Diseases Hospital and Research Centre (SIDH &RC), a 175 bedded dedicated first Infectious Diseases Hospital of Karachi, Pakistan. The hospital was established in July 2020. All patients >16 years of age who were admitted with a positive COVID PCR from July 2020–Mar 2022 were included. First COVID wave in Pakistan lasted from Mar-July 2020, second wave from Oct 2020 to Jan 2021, third wave from Mar-May 2021, fourth from July to Sept 2021 while fifth from Jan 2022 to Mar 2022. Demographic and clinical data was collected of patients from each wave and analyzed by SPSS to compare the difference amongst different COVID waves. Chi-square and t-test were used for analysis.

**Results:**

From July 2020-Mar 2022, 2900 COVID-19 patients were admitted. The total number of patients in each wave (from second to fifth waves) were 1217,783,590 and 310 respectively. The mean age in years was 60 in 2^nd^ and 3^rd^waves while it was 58 in 4^th^ and 66 in 5^th^ wave with 64.5%, 62.5%, 51.8%and 54% of males in each wave. On comparison majority 42% had moderate COVID in 2^nd^ wave, 37% and 48.5% had severe COVID in 3^rd^ and 4^th^ waves respectively while the disease was mild (31%) in 5^th^ wave (p-value of < 0.001).Cytokine releasing syndrome (CRS) was common in 3^rd^ wave (33%). Most intubations were done in 4^th^wave (21%). Compared to other waves mortality rate was also highest in 4^th^wave (35%, with p-valve of 0.03).

**Conclusion:**

Based on our data fourth wave which was mostly driven by delta variant was the deadliest in terms of disease category and outcome.

**Disclosures:**

**All Authors**: No reported disclosures.

